# Development and Validation of the Therapeutic Communication Scale in Nursing Students

**DOI:** 10.3390/healthcare12030394

**Published:** 2024-02-03

**Authors:** Soolgi Han, Jinhee Yoo, Kyonghwa Kang

**Affiliations:** 1 Department of Nursing, Kyungbok University, 425 Gyeongbok-daero, Jinjeop-eup, Namyangju-si 12051, Republic of Korea; soolgi84@gmail.com; 2 Department of Nursing, Kyung-in Women’s University, Gaehang-ro 96beon-gil, Jung-gu, Incheon 22319, Republic of Korea; olanzapine@hanmail.net; 3 Department of Nursing, Chungwoon University, 25 Daehakgil Honseongeup, Honseong 32244, Republic of Korea

**Keywords:** therapeutic communication, nursing student, psychometrics, patient-centered care

## Abstract

This study aimed to develop and validate a scale for measuring the therapeutic communication of nursing students. The scale development and evaluation study was conducted based on the scale development guidelines of DeVellis. A 47-item instrument was developed based on a review of the literature and interviews with 16 experts. Content validity was evaluated by ten nursing scholars, and the number of items was reduced to 35. The scale was administered to 352 nursing students from three regions in South Korea in 2022. Exploratory factor analysis and confirmatory factor analysis were performed on the scale items. Convergent validity, discriminant validity, and internal consistency reliability of the scale were evaluated. The factor analysis resulted in 15 items and two factors: relationship building and problem solving. Confirmatory factor analysis and evaluation of convergent and discriminant validity provided support for the validity of the two-factor Therapeutic Communication Scale in nursing students. The total scale demonstrated good internal consistency, with a McDonald’s omega (ω) of 0.89. The Therapeutic Communication Scale is a reliable and valid measure that can be used to assess nursing students’ therapeutic communication competence related to patient-centered nursing and provide foundational data to improve such skills.

## 1. Introduction

Therapeutic communication can be defined as targeted and focused conversations that identify thoughts, feelings, attitudes, and state changes related to health care according to the needs of the patient and assist in planning or revising treatments to achieve a state of well-being [1,2,3,4]. Therapeutic communication plays a vital role in forming a positive relationship with the patient to enhance the therapeutic effect [5,6]. Through therapeutic communication, patient satisfaction and safety are improved, and it has a positive impact on patient treatment outcomes, so it is very important for nursing students to embody this in the process of becoming a nurse [5,7,8]. This is an essential concept in nursing that encompasses the importance of interpersonal relationships, information transfer processes, clinical competencies, and patient-centered care [9]. However, nursing students often find it difficult to implement therapeutic communication because it is not visible.

Nursing students have the desire to act as a therapeutic tool, but they are worried about harming patients or interfering with treatment and express having difficulties with interacting with patients [4]. In clinical practice, there are few opportunities to communicate with patients, making it challenging for nursing students to acquire the necessary skills, which can contribute to a lack of confidence and fear of communicating [10]. While nursing education has included therapeutic communication theory, simulation-based communication training is a relatively new teaching method that is limited by some aspects. Therefore, multifaceted efforts are required by nursing colleges to produce nurses capable of fulfilling complex nursing requirements [11,12], including therapeutic communication.

The key elements of therapeutic communication include introducing oneself, forming relationships with patients, listening, empathy, respect, empathizing, confirming issues, and reconfirming [1,13]. However, the definition of therapeutic communication varies among scholars, making it difficult to measure its competencies [6,14]. Communication scales for medical professionals primarily emphasize information provision, accuracy, and directness [15]. Also, communication scales for medical professionals include opening of the conversation, establishing rapport, clarifying the interview, understanding the patient’s perspective, discussing the physical examination, and concluding the interview [16,17,18]. These are the important concepts for medical students to practice medicine as future doctors. In the GITCS (Global Interprofessional Therapeutic Communication Scale), a tool used to measure therapeutic communication among nurses, empathy, power sharing, and trust and rapport building are considered important. However, items such as ‘Provides balanced time on psycho-social and clinical aspects of patient care depending on the context’ [19] may not be appropriate for measurement among nursing students. For nursing students, the scale focuses on the elements of therapeutic communication, such as forming a relationship with the patient, understanding the patient’s perspective, and encouragement [20]. There are also tools that include education and feedback in their measurements [19]. Additionally, there are tools that measure the attitudes and behaviors of nurses during communication, but these tools focus on aspects such as the relationship with the patient and understanding the patients [21,22]. Such measures can be said to only apply to some aspects of the attributes of therapeutic communication, focusing on the technical part of the measurement. In the case of the Nursing Students’ Therapeutic Communication Questionnaire (NSTCQ), which measures therapeutic communication among nursing students, the attributes include promoting patient participation, maintaining dignity, preparedness, empathic understanding, and responsiveness [23]. However, upon closer inspection of the items, there are questions that check whether the student has studied, shared personal stories with the subject for sympathy, or properly assessed the patient. These aspects make it challenging to consider them as measurements of therapeutic communication. While therapeutic communication is emphasized in patient-centered nursing, it was difficult to find a communication scale measuring therapeutic communication competency.

Although there are tools that measure therapeutic communication skill, they are subject to limitations when it comes to measuring competencies in therapeutic communication among nursing students. Therefore, we aimed to develop a scale to assess nursing students’ therapeutic communication and verify its validity and reliability.

## 2. Materials and Methods

### 2.1. Study Design

**Figure 1:**
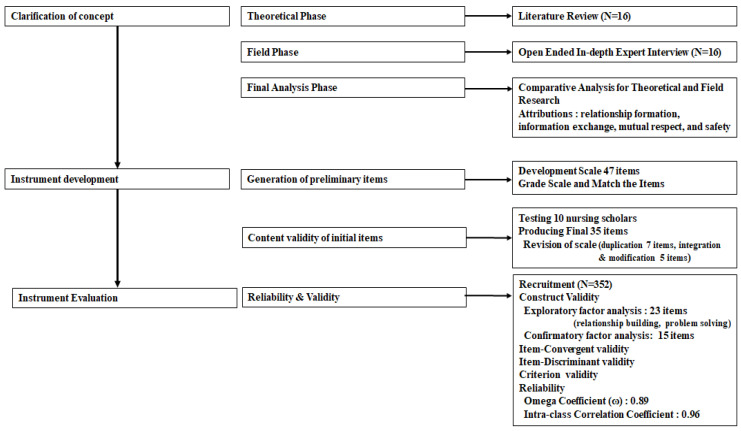
Steps in the development and evaluation of the Therapeutic Communication Scale.

This methodological study evaluated the reliability and validity of a new scale developed to assess therapeutic communication in nursing students. The Therapeutic Communication Scale (TCS) was developed as shown in Figure 1.

### 2.2. Scale Development

This study was performed in four stages according to the revised scale development of DeVellis [24]: (1) clarification of concept, (2) generation of preliminary items, (3) content validation of initial items, and (4) evaluation of validity and reliability.

#### 2.2.1. Clarification of Concept

This study was conducted to confirm the concept using the hybrid model of Schwartz-Barcott et al. [25]. The concept was identified by integrating the results of the theoretical phase through the literature review and the field phase involving expert interviews. The attributes and concept of therapeutic communication were confirmed through dictionary definitions and 16 research papers. In the field of nursing, communication tools were referenced from five papers [13,19,23,26,27], and for the conceptual aspects (definitions), four papers were referenced [6,9,13,28]. Through this, the situational dimensions (patient-centered, interactive, and adaptive factors) and technical dimensions (problem-solving factors) were identified, and three attributes of relationship formation, information exchange, and mutual respect were confirmed. The attributes of interactive factors include empathy, environmental control, and boundary setting [23,29,30,31], while adaptive factors include awareness, sensitivity, and self-reflection [23,32,33,34]. Problem-solving factors encompass active listening, silence, honesty, and clarity [2,9,20], and patient-centered factors include providing sufficient time and consideration [19,29,35,36,37]. The field study was conducted by 13 nursing professors with more than seven years of clinical and educational experience and three incumbent nurses with more than ten years of clinical experience and a master’s degree in nursing (Appendix B). The nursing professors participated in six focus group interviews with 2–3 people through an online platform, and the nurses were interviewed individually. The interview lasted from 1 h to 1 h 30 min. Through this process, one attribute of safety was added.

#### 2.2.2. Generation of Preliminary Items

Based on the identified attributes, existing tools, and expert interview data, 47 preliminary items regarding therapeutic communication were developed. A total of 16 items are included corresponding to relationship formation, 18 to exchanging information, 9 to respecting each other, and 2 to safety. Each item has a 4-point rating scale, with responses ranging from 1 (strongly disagree) to 4 (strongly agree). Higher scores indicate a higher level of therapeutic communication.

#### 2.2.3. Initial Content Validity Items

Content validity was assessed using ten nursing scholars: three professors of psychiatric nursing interested in therapeutic communication and with clinical experience working on a psychiatric ward and seven professors in the department of nursing with more than five years of work experience with patients and their guardians. Responses to items were recorded on a 4-point scale, where 1 = not at all valid and 4 = very valid. The results indicated no item had a content validity index less than 0.80. The percentage of each item receiving 3 or 4 points from experts was calculated, and the items with an average item level content validity index (CVI) of 0.8 or higher were selected as scale items [38]. There were no items with an average item-level CVI of less than 0.8. Seven items were deleted because they either duplicated another item or the meaning of the item was unclear. Five items were integrated, and their expressions were modified. Finally, this process resulted in 35 items that were selected. After discussions with the research team, a second opinion was sought from three nursing scholars who verified the validity of the content. In addition, grammatical errors, accuracy of expressions, and readability of the questionnaire items were checked by a professional with a doctorate in linguistics, confirming the final 35 items of the scale.

### 2.3. Scale Evaluation

#### 2.3.1. Study Samples

This study was conducted with the approval of the Institutional Review Board of C University (1041566-202103-HR-001-01). The participants were nursing students. The inclusion criteria were as follows: (1) those who had experienced at least two semesters of clinical nursing practice and (2) those who understood the purpose of the study and voluntarily agreed to participate. It was explained to the students who did not consent to participate in the study that there would be no personal disadvantages or additional risk factors (see Supplementary Material). The sample for the study used convenience sampling. The number of participants was based on the criterion that at least 150–200 participants are required for exploratory factor analysis (EFA) [39] and 150 or more participants are required for confirmatory factor analysis (CFA) [40]. A total of 352 nursing students participated in the study; therefore, the sample size was considered sufficient. After assigning a number to the collected questionnaires, they were randomly assigned using the random selection method in the SPSS Win 23.0 program, with the allocation ratio set to 50.0%. Participants were assigned to the EFA and CFA: 176 were used for the item analysis and EFA (Group 1), and 176 were used for the CFA (Group 2). The motivation for choosing a major, communication level, and satisfaction with the major of the two groups of participants were identified.

#### 2.3.2. Study Instruments

We measured interpersonal communication competence and communication self-efficacy to confirm the convergent validity of the TCS. Permission to use each scale in the study was granted via email from the original scale developer and the researcher who adapted the instrument.

##### Interpersonal Communication Competence

The Global Interpersonal Communication Competence Scale (GICC) developed by Rubin and Martin [20] and modified by Hur [41] was used to assess interpersonal communication competence (ICC). The scale consists of 15 items representing the following 15 factors: empathy, self-disclosure, social tension relief, concentration, assertiveness, interaction management, expressiveness, immediacy, support, efficiency, social appropriateness, comprehension, goal insight, responsiveness, and noise control. Each item is responded to using a 5-point scale, where 1 = strongly disagree and 5 = strongly agree, with higher scores indicating higher interpersonal communication competence. Cronbach’s α was 0.72 in Hur’s study [41], and Cronbach’s alpha was 0.75 in this study. A hypothesis for convergent validity in this study was that there would be a moderate correlation between the ICC and TCS scores.

##### Communication Self-Efficacy

The Korean version of the Self-Efficacy Questionnaire (KSE-120) was used to assess clinical communication skills (CSE) [42]. The KSE-12 has 12 items that are responded to using a 10-point Likert scale (1 = very uncertain, 10 = very certain). The total score can range from 12 to 120, and the higher the score, the higher the communication self-efficacy. A previous study reported a Cronbach’s alpha of 0.98 [42] , and Cronbach’s alpha was 0.98 in this study. The CSE was used to test the criterion validity of the TCS. In this study, it was hypothesized that the TCS would be moderately positively correlated with the CSE, since the CSE is generally known to be moderately correlated with TCS instruments [9].

### 2.4. Data Collection

Data were collected from January to February 2022. The participants were 3rd and 4th year nursing students enrolled in nursing departments at C University in Chungcheongnam-do, K University in Incheon, and K University in Gyeonggi-do, Republic of Korea. Nursing students who agreed to participate in the study were informed of the Google platform address for the online survey, along with a notice by a third party. Prior to data collection, cooperation was obtained from the directors of the three nursing departments. Data were collected only when the participants responded that they voluntarily agreed to participate in the survey without any external pressure. Additionally, it was emphasized that there was no disadvantage if they chose not to participate. Mobile gift icons were provided in return for participating in the survey. A total of 357 nursing students responded; however, five questionnaires were omitted because of duplicate responses. Therefore, 352 (98.6%) valid questionnaires were included in the analysis. A test–retest was conducted three weeks after administering the initial survey.

### 2.5. Statistical Analysis

IBM SPSS statistical version 25 and AMOS 25 were used for the analysis of data. An independent-samples *t*-test and chi-square test were conducted to verify the differences in general characteristics between the CFA and EFA groups. Items with an absolute value of 2 or more for skewness and kurtosis were deleted [43]. Items–total correlations that were above 0.30 were regarded as acceptable. After confirming that the Kaiser-Meyer-Olkin (KMO) was 0.8 or higher and the chi-square value of the Bartlett sphericity test was statistically significant (*p* < 0.05) [43], the factor structure was analyzed using the maximum likelihood method and oblimin rotation, a rectangular rotation method that assumes a correlation between factors [44]. CFA was conducted with Group 2 to verify the construct validity of the scale. To evaluate the model fit, the comparative fit index (CFI), Tucker–Lewis index (TLI), and root mean square error of approximation (RMSEA) were used. The fit criteria were CFI ≥ 0.90, TLI ≥ 0.90, and RMSEA ≤ 0.10 [45,46]. The criteria for item removal were set to a factor loading of less than 0.5 or more than 0.95 and a cross-loading of less than 0.3, but items were deleted after discussion among researchers. MTMM analysis was performed to determine the convergent and discriminant validity of the items. The item-convergent validity was checked after controlling for overlapping items in each item and the factor to which the item belonged and checking whether the correlation coefficient with the factor for the remaining items was 0.40 or higher. The item-discriminant validity was confirmed by the difference between the correlation coefficient with the factor to which each item belongs and the correlation coefficient with other factors. Criterion validity was evaluated by correlating the TCS with the CSE. McDonald’s omega (ω) was used to evaluate internal consistency reliability using IBM SPSS statistical version 29. Test–retest reliability was examined using the intra-class correlation coefficient (ICC).

### 2.6. Ethical Considerations

This study was approved by the Institutional Review Board of C University. Nursing students were asked to read an explanation of the study, including the purpose of the study, time required to participate, confidentiality of participation, benefits and disadvantages of participating in the study, and voluntary participation in the study. Students who provided consent to participate in the study were administered the survey questionnaire.

## 3. Results

### 3.1. Homogeneity Verification for Participants’ Characteristics

There were no statistically significant differences in the characteristics of Groups 1 and 2 with regard to motivation for choosing a major, level of communication skill, and satisfaction with the major (Table 1).

### 3.2. Item Analysis

There were no items whose absolute value of skewness and kurtosis were more than 2. Furthermore, it was confirmed that the item–total correlations by Pearson correlation of 35 items were more than the criteria (r ≥ 0.30), so they were included in the validity test, without any items deleted.

### 3.3. Construct Validity

#### 3.3.1. EFA

Prior to the EFA, the KMO was 0.93, suggesting that common latent factors existed among the items. Bartlett’s sphericity test χ^2^ value was 2253.89 (*p* < 0.001), confirming that the sample was appropriate for factor analysis. The criteria for item removal were set to a factor loading of less than 0.5 or more than 0.95 and a cross-loading of less than 0.3, but items were deleted after discussion among researchers, considering interpretability. The number of items in each subfactor was based on a minimum of three. As a result of the first exploratory factor analysis, there were 11 items in Factor 1 that were deleted due to factor loadings of less than 0.5, but 2 items (I meet clients without prejudice, I explain I can’t approve unreasonable demands of the client) were retained as they were, considering the opinions of the advisory panel. In Factor 2, there were four items, but one item (I give enough time for the client to organize their thoughts.) was retained as it was, taking into account the advisory panel’s opinion. As a result of the second exploratory factor analysis, 15 items in Factor 1 and 8 items in Factor 2 were retained, making a total of 23 items. The EFA indicated two factors had an eigenvalue exceeding 1, and the cumulative variance was 45.6%. Factor 1 consisted of 15 items and accounted for 37.7% of the variance. Factor 2 consisted of eight items and accounted for 7.9% of the variance. Factor 1 was named relationship building, and factor 2 was named problem solving. Considering the reduction in Eigenvalues when transitioning from two factors to three factors in the scree plot, it was deemed reasonable to consider it as two factors. The items also showed a value of factor loading of more than 0.5 in the two factors.

The results of the EFA are shown in Table 2.

#### 3.3.2. CFA

CFA was conducted to verify the model fit of the TCS factors extracted through EFA. First, the CFA satisfied the assumption of univariate normality in the item analysis. The relationship between variables and the goodness of fit were estimated using the maximum likelihood method, which calculates the loading of factors. The skewness ranged from −0.19 to 0.01, and the kurtosis ranged from −219 to 0.39, satisfying the skewness and kurtosis criteria. The first CFA goodness-of-fit indices were χ^2^ = 402.39, df = 229 (*p* < 0.001), TLI = 0.86, CFI = 0.88, and RMSEA = 0.07, indicating the model was not suitable.

Subsequently, the *p* value of the regression coefficient, standardization coefficient, variance of measurement error, and multiple correlations were checked, and measurement variables were removed if they did not meet the standard while considering interpretability. In the model, all *p* values of the regression coefficients were significant (*p* < 0.001), indicating a relationship between the latent and measured variables. However, item 6 was deleted because the standardized coefficient and factor loading were below standards. The measurement error variance was positive, and there was no Heywood case, but the model fit still did not meet the criteria (GFI = 0.857, TLI = 0.882, and CFI = 0.895).

Next, multiple correlations were identified. Because the latent variable is judged to explain the measured variable well only when the multi-correlation value is 0.40 or higher, if the multi-correlation value was less than 0.40, the item was deleted. In other words, considering model fit and interpretability, items with low multi-correlation coefficients whose explanatory power did not reach the criterion of 0.40 were sequentially deleted. Through this process, six items were deleted. As a result, the fit of the measurement model of 15 items with two factors showed an improvement in the fit indices compared to the model of 23 items. The model fit indices of the 15-item TCS were χ^2^ = 141.56, df = 89 (*p* < 0.05), GFI = 0.91, RMSEA = 0.06, CFI = 0.94, and TLI = 0.93. The index value was higher than 0.90 for the CFI and TLI and less than 0.10 for the RMSEA, which were the standard criteria. Therefore, the model appeared to agree with the measured data (Table 3).

The final version of the TCS consisted of two factors and 15 items (Appendix A). Factor 1 was relationship building and included nine items: #14 (empathy/expressing concern), #15 (empathy/behavioral cues), #4 (patient-centered environment/support), #20 (reaffirmation), #10 (comfort/adequate voice), #24 (giving information/feedback), #19 (noticing), #11 (accepting), and #25 (setting limitations). Factor 2 was problem solving and included six items: #12 (caring/professional attitude), #21 (factual), #29 (clarification), #17 (listening), #28 (truth/honesty), and #2 (giving enough time) (Table 4).

#### 3.3.3. Item-Convergent and Item-Discriminant Validity

The convergent validity of an items is the correlation coefficient between each item and the factors to which the item belongs. In this study, it was found to be 0.47~0.75, confirming that all items were satisfactory (cut-off of 0.40). The discriminant validity of the items was confirmed to be satisfactory, as the correlation coefficients for all items with the relevant factor were greater than those with other factors (Table 5).

#### 3.3.4. Criterion Validity

As the predefined hypothesis, the TCS was moderately correlated with the CSE (r = 0.64, *p* < 0.001), providing support for criterion validity. As hypothesized, the TCS score was correlated with the ICC, but the correlations were only moderate (r = 0.66, *p* < 0.001) (Table 6).

### 3.4. Reliability Evaluation

The results of the evaluation of the reliability of the TCS are provided in Table 4. McDonald’s omega (ω) of the total items of the TCS was 0.89:0.84 for factor 1 and 0.81 for factor 2. Test–retest reliability was evaluated by administering the questionnaire a second time to 95 participants after a period of three weeks. The ICC was 0.96 (95% CI [0.90–0.97]).

## 4. Discussion

This methodological study aimed to validate the scale of therapeutic communication in nursing students. This study reflected the therapeutic communication necessary for treatment with patients in the nursing education field as a scale and followed a systematic methodological process to establish the reliability and validity of the scale. Additionally, this scale has 15 items, so it has the advantage of being able to measure the therapeutic communication of nursing students easily.

The results of the factor analysis and a detailed discussion are presented as follows.

The exploratory factor analysis results showed that among the total 15 items of the TCS, the factor loadings of 12 items were adequate, ranging from 0.50 to 0.77. However, two items of relationship formation (#11, accepting; #25, setting limitations) and one item of problem solving (#2, giving enough time) had lower factor loadings, ranging from 0.42 to 0.49. Lower factor loadings imply lower explanatory power for the factor, indicating a lower degree of conceptual consistency with other variables within the factor, thus allowing for the possibility of factor deletion [47]. However, since CFA (confirmatory factor analysis) builds a model based on theory to confirm factor structure, items with factor loadings below 0.50 are still considered important for theoretical validation and acceptable if the factor loading is above 0.30 [46]. Previous studies [13,23,29,30,35] also support this concept as being important in therapeutic communication. To date, therapeutic communication in nursing students has emphasized empathetic listening and support, suggesting that future therapeutic communication by nursing students should be supplemented with aspects of giving patients time and being more accepting or setting limits.

Factor 1, which is the relationship formation factor, explained 37.67% of the variance, which was higher compared to other factors. When compared to the time of tool development, this factor was previously named the interactive factor and adaptive factor. Previous studies have also indicated that therapeutic communication is an essential skill for nursing students in forming interpersonal relationships [48]. Also, the Medical Communication Competence Scale (MCCS) presents socioemotional communication as a key attribute in communication [16], and the GICC tool shows similar results, with items such as ‘noticing not only what the conversation partner says but also what they do not say’ in the attribute of goal detection [20]. Additionally, in the NSTCQ, which measures therapeutic communication among nursing students, empathic understanding is presented as an important attribute of therapeutic communication [23]. Most tools measuring communication emphasize empathy, attitude, and relationship formation [19,30]. This is consistent with the components and attributes of therapeutic communication derived from the results of this study. However, the item ‘Explains that unreasonable demands cannot be accommodated’ regarding boundary setting is presented in the NSTCQ as well but without specific situations described for boundary setting, such as ‘I maintain professional boundaries in patient care’ [23]. Also, the attribute of boundary setting is not included in comprehensive communication [20], indicating a difference from therapeutic communication. Additionally, a distinctive feature of this study is that it includes items that recognize the ‘change’ in the state of the clients. Particularly, this change encompasses not only physical changes but also the state of nonverbal aspects. It also provides evidence that therapeutic communication is a tool for provision of health care through empathy and by respecting interpersonal boundaries [49]. Relational formation factors have been mentioned in previous studies targeting nurses, but factors related to environmental aspects, such as creating a comfortable atmosphere for conversation, were omitted [26,50]. In this study targeting nursing students, it was confirmed that environmental factors are included and significantly addressed in the formation of relationships during therapeutic communication.

Factor 2, the problem-solving aspect, involves focusing on the patient’s problems and providing ample time for them to talk about their own issues. Additionally, it entails maintaining a professional attitude, listening carefully, not making assumptions, and engaging in fact-based, patient-centered communication. Therapeutic communication recognizes the verbal and nonverbal needs of the client in the process of solving their nursing problem and provides for a comfortable environment and situation to help solve the problem [51]. For this reason, therapeutic communication can be regarded as an essential element in the process of solving the nursing problems of clients. Among the therapeutic communication items presented in this study, “Ask questions one by one” and “Listen to the end without interrupting” can be used to collect information. “Do not make assumptions about the subject’s words” and “Do not give hasty advice or advice without the subject’s consent” can be utilized in feedback or evaluation to help the patient solve their problem. This aligns with the emphasis on problem solving found in existing literature [2,20,35]. In particular, this aspect is considered important not only in therapeutic communication but also in relation to clinical performance skills. However, in this study, we examined the overall content related to the problem solving of the clients, including methods of asking questions to check for changes in the clients and how to listen to the clients’ stories. This factor had an explained variance of 7.94% but an Eigenvalue of 1.83, which satisfied the selected condition when the Eigenvalue was greater than 1. In particular, a previous study [52] selected a factor when the explained variation was more than 6%. Efforts will be needed to refine the tool through additional research on a larger number of subjects in the future.

Convergent and discriminant validity were satisfied through MTMM. This indicates that each item has a high correlation with the items of the corresponding subfactor and is distinguished from the items of other subfactors, indicating that each subfactor measures unique properties of therapeutic communication. In other words, the two subfactors, relationship formation and problem solving, are closely related to therapeutic communication, and each subfactor can be seen to have unique properties.

The criterion validity of the TCS was verified with significant positive correlations with CSE. ICC also showed a significant positive correlation with TCS. This result means that the higher the communication self-efficacy, the higher the likelihood of accurately using therapeutic communication. In the future, nursing students should be educated to improve interpersonal skills and self-efficacy to improve patient safety and positive outcomes.

As a result of confirmatory factor analysis, χ^2^ confirms the degree of agreement between the model and data, and the significance level must be greater than 0.05. The CFI and TLI, which indicate how well the model explains the entire dataset, were above 0.90, and RMSEA was below 0.10. These results are satisfactory for most criteria, so the model fit can be considered to be at a relatively appropriate level. Therefore, this scale can validly evaluate the therapeutic communication of nursing students according to the results of content validity, construct validity, and convergent and discriminant validity.

In instrument reliability verification, the internal consistency omega coefficient (ω) of the TCS subfactors ranged from 0.81 to 0.84, and the overall instrument reliability was excellent, at 0.89. These results show that it is a reliable tool with internal consistency supported.

The results of this study support the reliability and validity of the TCS as a measure that can be used to assess therapeutic communication skills in nursing students in the form of a self-report questionnaire and to evaluate the level of improvement in such skills subjectively. The items include behaviors, cognitions, and coping strategies that clinical nurses should possess; it is expected that they will be used in the future not only at school sites but also at clinical sites. However, in the case of an educational evaluation, as a self-report measure, the scores represent a subjective appraisal of learning outcomes. Therefore, an objective appraisal should be used to complement the self-report assessment when evaluating student performance. A limitation of this study is that the scale developed for nursing students should have been reviewed for face validity by nursing professors with experience in theoretical understanding and application of therapeutic communication. Future research will be needed to supplement the Therapeutic Communication Scale by collecting the opinions of nursing students. Lastly, based on the concepts and properties identified in this study, we hope that it will be used in a variety of ways, such as to help in developing therapeutic communication education programs for nursing students and in measuring educational effectiveness.

## 5. Conclusions

This study is scale development research aimed at assessing therapeutic communication, a conceptually ambiguous concept, among nursing students. It involved a review of domestic and international literature, expert interviews with nursing college professors, and content validity verification by expert groups. Through this process, the Therapeutic Communication Scale (TCS) was developed. The developed tool was validated for construct validity, convergent validity, and discriminant validity, and its reliability was also verified. As a result, it was finalized as a survey tool with two factors (relationship-centered communication and problem-solving-centered communication), 15 items, and a 4-point Likert scale. The TCS has been shown to be a valid and reliable tool for assessing therapeutic communication in nursing students. Confirming the concept of therapeutic communication and developing a scale is a significant result in understanding therapeutic communication among nursing students. The changing nursing environment is reflected in the items, and it is expected to positively impact the ability of nursing students to provide patient-centered care during clinical practice. In the future, it can be used as an effective tool for assessing the therapeutic communication of nursing students before and after therapeutic communication.

## Figures and Tables

**Table 1:** Homogeneity test between the two groups (N = 352).

Variable		Group 1 n (%) or Mean ± SD	Group 2 n (%) or Mean ± SD	χ^2^/t	*p*
Age		25.24 ± 7.26	25.92 ± 7.90	−0.84	0.400
Gender	Female	165 (93.9)	163 (92.6)	0.18	0.833
	Male	11 (6.3)	13 (7.4)		
Religion	Catholic	34 (19.3)	40 (22.7)	1.09	0.780
	Christian	20 (11.4)	23 (13.1)		
	Buddhist	14 (8.0)	14 (8.0)		
	Other	108 (61.4)	89 (56.3)		
Motivation for choosing a major	Voluntary	146 (83.0)	159 (90.3)	4.15	0.059
	Recommendation	30 (17.0)	17 (9.7)		
Level of communication skill	Very good	21 (11.9)	11 (6.3)	4.42	0.220
	Good	79 (44.9)	76 (43.2)		
	Common	62 (35.2)	75 (42.6)		
	Lacking	14 (8.0)	14 (8.0)		
Satisfaction with major	Satisfied	161 (91.5)	163 (92.6)	0.16	0.844
	Dis-satisfied	15 (8.5)	13 (7.4)		

Note: The sample size for each group was 176.

**Table 2:** Exploratory factor analysis of the Therapeutic Communication Scale.

Item	Factor	
	1	2
14. I verbalize about clients’ situation or how they feel.	0.77	0.23
5. I focus my conversation on what the patient wants to know.	0.75	0.24
1. I introduce myself to the patient with a greeting appropriate to the situation and atmosphere.	0.73	0.17
15. I express how I understand the client by gestures and eye contact.	0.71	0.19
7. I express my willingness to help patients in need.	0.71	0.22
4. I build a comfortable atmosphere for conversation.	0.70	0.21
20. I recheck clients’ words and behaviors when they are not clear.	0.59	0.18
10. I use an appropriate tone and volume when I talk.	0.57	0.41
24. I give information easy enough for the client to understand.	0.56	0.34
32. I explain nursing interventions in an easy-to-understand manner so the patient can understand them.	0.56	0.16
8. I match my words and facial expressions.	0.53	0.28
19. I notice changes in body language, facial expressions, and emotions even if the patient does not speak.	0.52	0.22
6. Even when feeling tension, I calmly deal with patients.	0.50	0.32
11. I meet clients without prejudice.	0.46	0.39
25. I explain I can’t approve unreasonable demands of the client.	0.42	0.23
23. When talking to my patient, I make up topics as I please.	0.43	0.77
12. I don’t give advice or recommendations without clients’ approval.	0.14	0.62
21. I don’t assume what the client will say.	0.21	0.59
29. I only ask the patient one question at a time.	0.35	0.57
17. I listen to the patient’s speech until the end without interrupting or blocking.	0.11	0.57
18. While listening to the person’s story, I do not think in advance about the following question to ask.	0.01	0.53
28. I don’t reduce or exaggerate the clients’ problem.	37	0.52
2. I give enough time for the client to organize their thoughts.	0.33	0.49
Eigenvalue	8.66	1.83
Explained variance (%)	37.67	7.94
Cumulative variance (%)	37.67	45.61

Kaiser-Meyer-Olkin KMO = 0.92, Bartlett’s sphericity χ^2^ = 1669.59, df = 253, *p* < 0.001. The background color represents the categories.

**Table 3:** The changes in goodness of fit for the Therapeutic Communication Scale.

Model	χ^2^ (*p*)	df	GFI	CFI	TLI	RMSEA
2 Factors, 23 Items	402.39 (<0.001)	229	0.84	0.88	0.86	0.07
2 Factors, 15 Items	141.56 (<0.05)	89	0.91	0.94	0.93	0.06

GFI = goodness-of-fit index; CFI = comparative fit index; TLI = Tucker–Lewis index; RMSEA = root mean square error of approximation.

**Table 4:** Confirmatory factor analysis of the Therapeutic Communication Scale.

Factor	Item	Standardized Estimate (β)	SE	Critical Ratio	*p*	AVE	Omega (ω)	ICC
1	14	1.00				0.92	0.84	0.96
	15	0.92	0.11	8.76	<0.001			
	4	0.90	0.12	7.36	<0.001			
	20	1.07	0.13	8.52	<0.001			
	10	0.97	0.12	8.32	<0.001			
	24	1.16	0.13	9.01	<0.001			
	19	1.03	0.13	7.92	<0.001			
	11	0.83	0.14	5.87	<0.001			
	25	0.92	0.15	5.99	<0.001			
2	2	1.00				0.91	0.81	
	28	1.53	0.24	6.37	<0.001			
	17	1.37	0.23	6.02	<0.001			
	29	1.31	0.23	5.68	<0.001			
	21	1.49	0.24	6.15	<0.001			
	12	1.42	0.23	6.17	<0.001			
Total scale							0.89	

SE = standard error; AVE = average variation extracted; ICC = intra-class correlation coefficient.

**Table 5:** Item-convergent and item-discriminant validity.

Factor	Items	Relation Building	Problem Solving
Relation building	14	**0.75**	0.52
	15	**0.68**	0.36
	4	**0.58**	0.40
	20	**0.66**	0.51
	10	**0.65**	0.39
	24	**0.69**	0.48
	19	**0.62**	0.37
	11	**0.47**	0.14
	25	**0.48**	0.16
Problem solving	2	0.07	**0.52**
	28	0.51	**0.74**
	17	0.41	**0.66**
	29	0.35	**0.59**
	21	0.49	**0.69**
	12	0.45	**0.69**

Bold numbers indicate meaningful item-convergent validity.

**Table 6:** Correlations between the Therapeutic Communication Scale and other scales.

Item	Factor 1 (Relation Building) r (*p*)	Factor 2 (Problem Solving) r (*p*)	TCS r (*p*)
ICC	0.69 (<0.001)	0.47 (<0.001)	0.66 (<0.001)
CSE	0.58 (<0.001)	0.57 (<0.001)	0.64 (<0.001)

ICC = Interpersonal Communication Competence Scale; CSE = Communication Self-Efficacy Scale; TCS = Therapeutic Communication Scale.

## Data Availability

Data will be made available upon request.

## Supplementary Materials

The following supporting information can be downloaded at: https://www.mdpi.com/article/10.3390/healthcare12030394/s1, Communication competency assessment for nursing students.

## Appendix A. Therapeutic Communication Scale in Nursing Students

**Item**		**Strongly** **Agree**	**Agree**	**Disagree**	**Strongly** **Disagree**
1	I verbalize about clients’ situation or how they feel.	4	3	2	1
2	I express how I understand the client by gestures and eye contact.	4	3	2	1
3	I build a comfortable atmosphere for conversation.	4	3	2	1
4	I recheck clients’ words and behaviors when they are not clear.	4	3	2	1
5	I use an appropriate tone and volume when I talk.	4	3	2	1
6	I give information easy enough for the client to understand.	4	3	2	1
7	I notice changes in body language, facial expressions, and emotions even if the patient does not speak.	4	3	2	1
8	I meet clients without prejudice.	4	3	2	1
9	I explain I can’t approve unreasonable demands of the client.	4	3	2	1
10	I don’t give advice or recommendations without clients’ approval.	4	3	2	1
11	I don’t assume what the client will say.	4	3	2	1
12	I only ask the patient one question at a time.	4	3	2	1
13	I listen to the patient’s speech until the end without interrupting or blocking.	4	3	2	1
14	I don’t reduce or exaggerate the clients’ problem.	4	3	2	1
15	I give enough time for the client to organize their thoughts.	4	3	2	1

## Appendix B. General Characteristics of Participants in Expert Interview

**Variable**		**n (%)**	**Mean ± SD**
Gender	Female	16 (100.0)	
	Male	0 (0.0)	
Age (years)	≤45	4 (25.0)	46.73 ± 5.24
	≥46	12 (75.0)	
Work department	College	13 (81.2)	
	Hospital	3 (18.8)	
Work experience	Psychiatric unit in tertiary hospital	7 (43.8)	
	General unit in tertiary hospital	5 (31.2)	
	General hospital	2 (12.5)	
	Community mental health facility	2 (12.5)	
Clinical nursing experience (years)	≤5	5 (31.2)	11.31 ± 7.3
	6–15	5 (31.2)	
	≥15	6 (37.6)	
Educational experience	No	2 (12.5)	
	Part-time lecturer	2 (12.5)	
	Less than 5 years as a nursing professor	5 (31.2)	
	More than 6 years as a nursing professor	7 (43.8)	
