# Supplementary material for: Development and Validation of the Therapeutic Communication Scale in Nursing Students
Source: Healthcare (Basel). 2024 Feb 3;12(3):394. doi: 10.3390/healthcare12030394 (PMC10855793; doi:10.3390/healthcare12030394)
Supplement: Supplementary file 1 [file healthcare-12-00394-s001.zip › healthcare-2775547-supplementary.pdf]

Development and Validation of the
Therapeutic Communication Scale in Nursing Students

Hello,

This questionnaire is intended for the development of a therapeutic communication measurement tool in practical nursing education. It takes approximately 10 minutes to complete the questionnaire, and the personal information you provide will only be used for research purposes. There are no personal disadvantages (such as in grading or employment) or additional risks associated with completing the questionnaire. Your confidentiality is assured, so please check for any missing items and complete the questionnaire in its entirety. If you have any questions related to this, please feel free to contact us at any time.

Researcher
Department of Nursing, Chungwoon University, Professor Kyongwha Kang (kh_kang@chungwoon.ac.kr)
Department of Nursing, Kyung-in Women's University, Professor Jinhee Yoo (palanca@kiwu.ac.kr)
Department of Nursing, Kyungbok University, Professor Soolgi Han (soolgi@kbu.ac.kr)

* I have read the above information and voluntarily participate in the research for the development of the communication measurement tool.
Agree □
Disagree □

◎ Guidance on Consent for the Collection and Use of Personal Information
1. Purpose of Collecting and Using Personal Information
   - Consent to participate in the survey.
2. Personal Information Items Collected
   - Name, student ID.
3. Retention and Use Period of Personal Information
   - Until the end of the course (February 28, 2022).
4. Right to Refuse Consent and Guidance on Disadvantages Due to Refusal
   - You have the right to refuse consent to the collecting and using of personal information, and there will be no disadvantages for refusing.

[Consent under the Personal Information Protection Act] I understand that it is necessary to use information about the survey participant's personal details for the purpose of receiving a token of appreciation after participating in the survey. I consent to provide various information materials related to myself, which are protected under the [Personal Information Protection Act], for this purpose.

I have read and agree to the above content □
